# Syringic acid, a promising natural compound for the prevention and management of metabolic syndrome: A systematic review

**DOI:** 10.22038/ijbms.2025.85940.18568

**Published:** 2025

**Authors:** Habibeh Mashayekhi-Sardoo, Fatemeh Sadeghzadeh, Maryam Rameshrad, Hossein Hosseinzadeh

**Affiliations:** 1 Bio Environmental Health Hazards Research Center, Jiroft University of Medical Sciences, Jiroft, Iran; 2 Student Research Committee Jiroft University of Medical Sciences, Jiroft, Iran; 3 Department of Pharmacodynamics and Toxicology, School of Pharmacy, Mashhad University of Medical Sciences, Mashhad, Iran; 4 Pharmaceutical Research Center, Pharmaceutical Technology Institute, Mashhad University of Medical Sciences, Mashhad, Iran

**Keywords:** Diabetes, Dyslipidemia, Hypertension, Metabolic syndrome, Obesity, Syringic acid

## Abstract

In recent years, because of the changes in modern lifestyles, the incidence of metabolic syndrome (MetS) has been increasing. MetS is a cluster of conditions, including hypertension, insulin resistance, hyperlipidemia, and obesity, that occur together, increasing the risk of cardiovascular diseases (CVDs), stroke, and type 2 diabetes. Therefore, this review provided comprehensive information on the protective effects of syringic acid (SYR) on the main components of MetS using natural phenolic acid compounds such as SYR. An exhaustive search was conducted using keywords related to SYR and MetS in scientific databases like Scopus, Web of Science, PubMed, and Google Scholar from inception to August 2024. The review included all *in vitro*, *in vivo*, and clinical research. Preclinical studies showed that SYR has protective effects against MetS, including diabetes, CVDs, dyslipidemia, and obesity. SYR has shown antidiabetic effects in animal models, lowering blood glucose and improving insulin levels. It also mitigated cardiac injury biomarkers, decreased oxidative stress, and improved lipid profiles. In animal models, SYR could lower body weight and fat mass, lower leptin levels, enhance adiponectin circulation, and improve insulin resistance. Nonetheless, there was insufficient clinical research on SYR’s influences on humans undergoing MetS. Further research, especially randomized controlled trials, is needed to examine its effectiveness, safety, optimal doses, and long-term effects.

## Introduction

**Figure 1:**
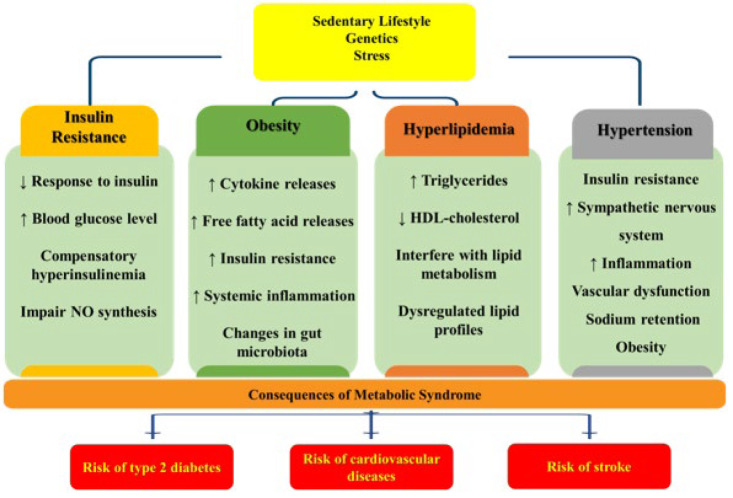
This schematic diagram shows the pathophysiology of metabolic syndrome

HDL: high-density lipoprotein; NO: nitric oxide

Around 5% of adolescents and 3% of children globally experienced Metabolic syndrome (MetS) in 2020 (1). MetS is a combination of risk factors, including insulin resistance, dyslipidemia, abdominal obesity, and hypertension, which can lead to diabetes mellitus, stroke, and cardiovascular diseases (CVDs) (2). Insulin resistance results in enhanced blood glucose levels and compensatory hyperinsulinemia progressing to type 2 diabetes (3). Obesity, especially visceral obesity, is an alarming risk factor, as the release of inflammatory cytokines and free fatty acids (FFA) from adipocytes (4). Hypertension is generally linked to MetS, as insulin resistance, obesity, and inflammation induce vascular dysfunction and elevated sympathetic nervous system function (5, 6). Chronic mild inflammation in adipose tissue deteriorates insulin resistance, triggering CVD risk (7). The pathophysiology of MetS involves different factors such as endothelial dysfunction, inflammatory pathways, gut microbiota alterations, and oxidative stress processes (8, 9). Finally, MetS may damage cells and lead to diabetes mellitus, stroke, and CVDs (Figure 1) (2).

According to Codazzi *et al*. (2024), MetS can also develop after additional risk factors, including genetic, environmental, and lifestyle factors (10). Unfortunately, MetS in childhood is a leading cause of MetS, atherosclerosis, and type 2 diabetes mellitus in adulthood (11) (Figure 1). Since MetS causes a high rate of disorders, numerous attempts have been made to prevent and treat MetS (12). 

The prevention and development of MetS are remarkably influenced by diet, which is a modifiable factor. A diet rich in phenolics, like the Mediterranean diet, can decrease the risk of cardiometabolic disorders (13). Owing to the (poly)phenols being effective donors of electrons or hydrogen atoms, they can stop the production of reactive oxygen species (ROS) and oxidative stress development in cells (14, 15). Hence, polyphenols are effective in managing diseases related to oxidative stress, especially diabetes and MetS (16). In addition to drug repositioning (17, 18), numerous studies have explored the effectiveness of plants or natural products in managing MetS components, such as pomegranate (*Punica granatum* L. ) (19), chamomile (20), saffron (21), walnut kernel (22), green tea (23), quercetin (24), oleanolic acid (25), caffeic acid (26), and ginsenosides (27, 28). 

The phytochemical syringic acid (SYR) has drawn the interest of researchers owing to its anti-oxidant and anti-inflammatory properties (29-31). Fruits and vegetables such as olives, pumpkin, acai palms, grapes, and black soybeans are rich sources of SYR, a phenolic acid derivative of hydroxybenzoic acid (31-33). Some therapeutic effects of SYR are anti-microbial (34), anti-apoptotic (29), anti-diabetic (35), and cardio and neuroprotective activities (31, 36-38). SYR, as a promising natural agent, may help prevent and control MetS through different molecular mechanisms. It contributes to beneficial effects via modulating genes involved in lipid metabolism, preventing fat cell formation (adipogenesis) (39), and inhibiting inflammation by the NF-κB and JAK-STAT pathways (40). Furthermore, it promotes anti-oxidant defenses via the Nrf2 pathway (41), increases insulin signaling through activating PI3K/Akt (42, 43), and provides specific protection to tissues such as the liver, heart, and brain.

Previously, Bartel *et al*. (2023) reviewed the effect of SYR on civilization diseases without particularly discussing obesity, dyslipidemia, and MetS (33). The present article comprehensively focuses on studies that investigated the protective effects of SYR on the main clusters of MetS, especially diabetes, hypertension, obesity, and hyperlipidemia, summarizes the cellular mechanisms *in vitro* and *in vivo*, and discusses the clinical findings. This article synthesizes evidence from these studies to highlight SYR’s potential as a therapeutic agent for MetS. Examining the effects of SYR on MetS symptoms can help develop new and effective treatment strategies.

## Methods

To write this review, a comprehensive search was performed using Scopus, Web of Science, PubMed (Medline), and Google Scholar databases from inception to May 2025. The following medical subject headings (MeSH terms) and free-text keywords were investigated alone or in combinations: “Syringic Acid”, “blood pressure”, “hypertension”, “anti-hypertensive”, “hypotensive”, “diabetes mellitus”, “diabetes”, “blood glucose”, “anti-diabetic”, “hyperglycemia”, “anti-hyperglycemic”, “hypoglycemic”, “insulin”, “dyslipidemia”, “hyperlipidemia”, “high cholesterol”, “hypercholesterolemia”, “high triglyceride”, “hypertriglyceridemia”, “atherogenic”, “atherosclerosis”, “cardiovascular disease”, “obesity”, “anti-obesity”, “overweight”, “weight loss”, and “appetite”. All *in vitro, in vivo*, and clinical studies were considered in this review article. This study also evaluated the research on the SYR used in polyherbal medicine or polyherbal formulations. Most focused articles were in English, and studies in other languages were assessed based on their English abstracts. Review articles, conference abstracts, and dissertations were excluded from our assessment. 

**Figure 2:**
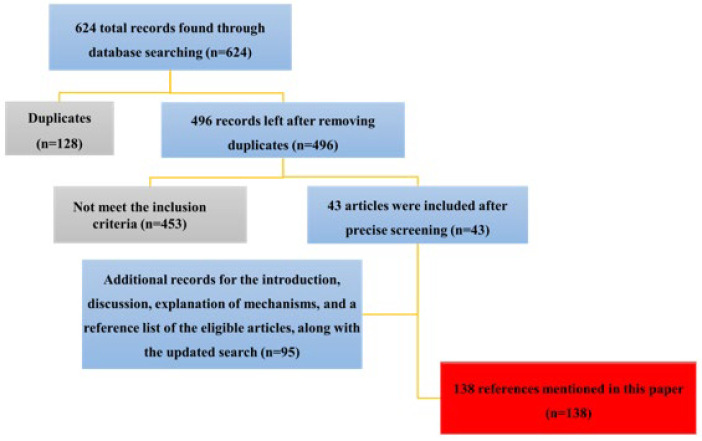
This schematic diagram shows the search strategy of the present study

Out of 624 relevant citations identified by the search strategy, 128 duplicate articles were deleted. The screening was completed by reading the titles, abstracts, or full texts; following the scrutiny screening, 43 articles were left. Moreover, the reference list of the eligible articles was searched for more results, and some articles were added to complete the introduction and explain the mechanisms; after completing the manuscript, an updated search was conducted to include newly published sources. These all included the addition of 95 records. In total, 138 articles were reviewed in this study (Figure 2), and they were classified into four main headings: insulin resistance, dyslipidemia, CVDs, and obesity. Each section summarizes the main findings from the *in vitro*, *in vivo*, and clinical research (if available), discusses the suggested mechanisms of function, and identifies areas needing more research. 

In addition, the current study provides tables summarizing the clear findings of the *in vitro* and *in vivo* studies, including data on the study models used for SYR doses or concentrations, and key findings. We present existing evidence for SYR’s protective influences on each MetS component. 

## Results and Discussion

### Effects of syringic acid on insulin resistance

Diabetes mellitus is a metabolic disorder that has increasingly posed a threat to human health over the past century, imposing huge economic, social, and human burdens (44-46). MetS increases the risk of heart disease, stroke, and type 2 diabetes (47). Type 2 diabetes mellitus arises from a deficiency of insulin or resistance to insulin, manifesting a rise in blood glucose levels (48, 49). Other risk factors associated with diabetes development include diet, obesity, body weight, physical activity, alcohol consumption, and smoking (50). Uncontrolled diabetes results in cardiovascular complications and dyslipidemia (51-53). To summarize, since MetS is linked to the progression of type 2 diabetes, improvements in the subset of MetS contributed to reducing the risk of diabetes (54).

We reviewed the effect of SYR on diabetes mellitus in clinical and preclinical research. Although we could not find clinical research about SYR on diabetes mellitus, we evaluated 16 articles in animal experimental models and 6 *in vitro* studies. Descriptive data for the studies included are listed in Table 1.

*In vivo studies*

Glucose is the chief source of energy requirement in cells for the continuation of life (55). The liver is the main organ in the adjustment of glucose homeostasis, and its function is disturbed during diabetes. Each alteration in the activity of enzymes implicated in glucose homeostasis results in the accumulation of glucose in cells, glucose toxicity, and finally, adverse side effects of diabetes (56). In the literature on finding natural sources for relieving diabetes, there seems to be general agreement that medicinal fruits and vegetables with high contents of phenolic acids prevent different diseases like diabetes (57). 

A recent study investigated the effects of SYR on hepatotoxicity and diabetes induced by sodium arsenite in mice. Thirty male mice were divided into five groups: control, SYR (25 mg/kg for the last week), sodium arsenite (3 mg/kg for 30 days), and two therapeutic groups receiving SYR (10 and 25 mg/kg for the last week). The results indicated that administering SYR before sodium arsenite exposure reduced liver enzyme levels, fasting blood sugar (FBS), glucose tolerance test (GTT) results, nitric oxide (NO), tumor necrosis factor-alpha (TNF-α), and thiobarbituric acid reactive substances (TBARS). Additionally, SYR treatment increased total thiol levels, catalase (CAT), superoxide dismutase (SOD), glutathione peroxidase (GPx), and caspase-3 expression. Based on these findings, SYR may be recommended as a treatment option to mitigate the hepatotoxic and diabetogenic effects of sodium arsenite by reducing oxidative stress, inflammation, and apoptosis (58).

Based on the Indian traditional prescription, three doses of SYR (25, 50, and 100 mg/kg; 30 days) were administered to alloxan (150 mg/kg; IP a single dose)-induced diabetic albino Wistar rats, and the function of crucial enzymes involved in carbohydrate metabolism was evaluated. The results showed that SYR increased glycogen, insulin, and hemoglobin levels while decreasing plasma glucose and glycated hemoglobin (HbA1c) in diabetic animals dose-dependently. Moreover, the histopathological findings revealed the effective role of SYR in reducing pancreatic damage following alloxan administration to rats and its role in the stimulation of the regeneration of cells and insulin secretion from residual β-cells. In addition, SYR restored the function of enzymes for carbohydrate metabolism, including glucokinase (GK) and glucose 6-phosphate dehydrogenase (G6PD). SYR also, via enhancing the nicotinamide adenine dinucleotide phosphate (NADPH) synthesis, decreased oxidative stress. Taken together, SYR, as a natural antidiabetic agent, protects pancreatic β-cells from injuries, improving insulin secretion, and increasing carbohydrate metabolism, managing the induced diabetes. However, more research to find the precise underlying anti-diabetic mechanism of SYR is suggested (59). 

In addition, this team in their previous experiment found that SYR (50 mg/kg) administration for 30 continuous days to alloxan (150 mg/kg; IP a single dose)-induced diabetic albino Wistar rats resulted in a decrease in plasma glucose levels and an increment in the amounts of plasma insulin and C-peptide in comparison to untreated rats. Interestingly, SYR returned the content of tissue and plasma glycoprotein (hexose, sialic acid, Fucose, and hexosamine) to near-normal ranges. It can be assumed that SYR concurrently exerts antidiabetic effects and improves the abnormalities of glycoprotein components. The underlying mechanism of SYR may include the modulation of glycoprotein components that are important in different physiological functions, such as cell signaling and immune response. Then, SYR can manage insulin sensitivity or enhance the metabolism of glucose in tissues. Overall, SYR, due to its anti-oxidant and anti-inflammatory effectiveness, aids in the management of diabetes and related complications. However, more research is required to find underlying mechanisms, optimal doses, and therapeutic applications in clinical settings (36). 

The theoretical framework underpinning Sahari *et al*.’s study assessed the effects of SYR on preserving the pancreas function and carbohydrate metabolism in diabetic rats. In the same vein, SYR (50 mg/kg; consecutive 60 days) was prescribed orally to Sprague-Dawley rats that were induced to diabetes by a single dose of Streptozotocin (STZ) (40 mg/kg; IP). Standard drug glimepiride (0.1 mg/kg) was considered as a positive control. Results obtained by the researchers showed that SYR significantly reduced blood glucose and HbA1C levels and improved insulin levels in diabetic rats compared to non-diabetic rats. SYR significantly modified the activity of enzymes involved in carbohydrate metabolism. It reduced the glucose-6 phosphatase and fructose 1,6-bisphosphatase activities but elevated the function of glycolytic enzymes, including hexokinase and pyruvate kinase in diabetic rats’ liver. Although both SYR and glimepiride groups improved glucose and HbA1c levels, SYR exhibited superior effects in increasing insulin amounts and regulating enzyme functions. In summary, the molecular-pharmacological basis of SYR is increasing glycolytic enzyme function, preventing gluconeogenic enzyme activity, and potentially improving the secretion of insulin. Hence, SYR as a potent candidate could promisingly manage glucose homeostasis in diabetic conditions. However, further studies are warranted to elucidate its precise mechanisms and therapeutic applications in diabetes management (60).

Sahari *et al*. in another study evaluated the protective effect of SYR on the pancreas of diabetic rats. Hence, SYR (50 mg/kg; consecutive 60 days) was administered orally to Sprague-Dawley rats induced by STZ (40 mg/kg; IP; a single dose). Standard drug glimepiride (0.1 mg/kg) was considered as a positive control. Findings indicated that SYR significantly reduced blood glucose and HbA1C levels and improved plasma insulin levels in diabetic rats compared to non-diabetic rats. Furthermore, SYR rehabilitated the anti-oxidant system in STZ-diabetic rats, thereby enhancing the activity of CAT, SOD, GPx, and glutathione reductase (GR) as anti-oxidant enzymes in their pancreas tissue. In short, due to its intrinsic anti-oxidant and antihyperglycemic properties, SYR highlighted protective effects on the pancreatic function of diabetic rats. Moreover, SYR exhibited similar antihyperglycemic and anti-oxidant effects as glimepiride (an antidiabetic drug) without any adverse effects, so it can be proposed as a promising alternative to synthetic commercial medicines (35).

A study was planned to evaluate the protective effects of SYR on diabetes in neonatal Wistar rats. Afterward, STZ (split 110 mg/kg; IP) was administered on 2 and 3 postnatal days to neonatal rats. Consequently, the neonatal diabetic rats received SYR (25 mg/kg and 50 mg/kg; oral) from the 8th until the 18th postnatal weeks. The key findings revealed that SYR significantly improved and lowered hyperglycemia, polyuria, polydipsia, polyphagia, and HbA1c levels compared to non-diabetic neonates. SYR treatment restored Na/K ATPase function in different tissues, which is crucial for preserving the balance of cellular ions and their function. The decline in activity of this enzyme can cause diabetic complications, and SYR, via enhancing its activity, may bring about protective effects. Moreover, SYR attenuated the formation of Advanced Glycation End-products (AGEs). Furthermore, SYR attenuated cardiac hypertrophy and relative organ weight, probably by a decrease in oxidative stress and related cell injury biomarkers. In conclusion, SYR by multifaceted effects, such as anti-oxidant and anti-inflammatory effects, as well as improvement in glycemic control, and restoration of cellular activities contributed to the attenuation of hepatic, renal, cardiac, and neuronal injuries caused by chronic hyperglycemia in animals. Therefore, it could be considered as an adjuvant treatment with synthetic antihyperglycemic drugs to minimize the adverse side effects and onset of tolerance of antidiabetic medicine (37).

A study aimed to determine the synergic anti-oxidative and antidiabetic effects of Zinc (II; Zn (II)) and SYR in diabetic rats. Hence, regarding diabetes induction, a single dose of 10% fructose plus 40 mg/kg STZ was intraperitoneally prescribed to male Sprague Dawley rats. After a week the diabetic rats received Zn (II) (15 mg/kg) or/and SYR (45 mg/kg) for 4 weeks. Likewise, the complexing Zn (II) and SYR exhibited more antidiabetic effects in diabetic animals than other groups treated with Zn (II) or SYR, indicating a synergistic effect. In addition, the complex declined hyperphagia and polydipsia and balanced the oxidant/anti-oxidant system in diabetic rats. In some cases, the efficacy of the complex was similar to metformin (as a positive control). To sum up, complexing Zn (II) and SYR through moderating the glycogen synthesis in tissues, Akt phosphorylation, sensitivity and secretion of insulin, and the activity of muscle hexokinase highlighted more antidiabetic efficacy. The findings supported more exploration of natural compounds in diabetes treatment, leveraging their synergistic impacts in promoting clinical outcomes (61).

Hyperglycemia results in diabetic peripheral neuropathy (DPN) in half of type 1 or type 2 diabetes patients (62, 63). DNP results from the progressive loss of peripheral nerve function, leading to physical disability, intermittent pain, and decreased quality of life (62). 

Since diabetes is a metabolic disease associated with mitochondrial impairment and oxidative stress, the neuroprotective impacts of SYR on the mitochondrial function and oxidative stress of the brain, sciatic nerve, and spinal cord in STZ-diabetic rats were evaluated for the first time. The diabetic Sprague-Dawley rats (STZ: 60 mg/kg; IP) received different doses of SYR (25, 50, and 100 mg/kg) for 6 weeks. The high dose of SYR significantly relieved memory, learning, and movement disturbed by diabetes. Moreover, 100 mg/kg of SYR increased the expression of Peroxisome proliferator-activated receptor gamma coactivator 1-alpha (PGC-1α) and Nuclear respiratory factor 1 (NRF-1) in diabetic rat brains. Furthermore, 100 and 50 mg/kg of SYR elevated the mitochondrial DNA copy number (mtDNA-CN) in the brain and spinal cord of diabetic rats, respectively. It is suggested that SYR, through enhancement in mitochondrial biogenesis, modulates mitochondrial homeostasis in the brain, sciatic nerve, and spinal cord. SYR not only reduces lipid peroxidation in the brain, sciatic nerve, and spinal cord, but it also decreases demyelination and inflammation in the sciatic nerves of diabetic animals compared to non-diabetic animals. In doing so, new insights into the roles of PGC-1α and NRF-1 in alleviating diabetic neuropathy were provided. In short, SYR indicated a potent molecular-pharmacological profile that could be leveraged as a therapeutic agent aimed at mitigating diabetic neuropathy and improving neuroprotection via its anti-oxidant, metabolic regulation, and mitochondrial biogenic effects. Further research is warranted to clarify underlying mechanisms and pharmacological applications in broader clinical settings (64).

Type 2 diabetes plays a part in chronic kidney disease development (65). Besides, much attention has been paid to the prevalence, incidence, and risk factors of diabetic nephropathy, and thus its effect on developing other complications in Type 1 diabetes patients (66). Likewise, diabetic nephropathy occurs following chronic uncontrolled hyperglycemia that causes damage to the microvascular of the kidney (67). Finding effective strategies for treating diabetic kidney disease deserves more research attention (65). Sherkhane *et al.* attempted to find a link between autophagy and perturbed redox homeostasis with diabetic nephropathy progression. To induce diabetes, STZ (55 mg/kg; IP) was administered to male Sprague-Dawley rats. Four weeks after the diabetes induction, treating the diabetic rats began with SYR at doses of 25 mg/kg and 50 mg/kg orally. This treatment continued for an additional four weeks. Unfortunately, diabetes leads to an increase in oxidative stress biomarkers and a decrease in Nuclear respiratory factor 2 (NRF-2) activity. Moreover, diabetes, by lowering the expression of light chain (LC) 3-IIB in the kidney of diabetic animals, inhibited the autophagy process. Nevertheless, SYR significantly modulated the kidney function of diabetic rats and returned the levels of serum creatinine (Cr), urine Cr, and blood urea nitrogen (BUN) to normal ranges. Furthermore, the molecular results indicated that SYR significantly improved the expression of autophagy-related proteins (Atg3, Atg5, and Atg7) and Nrf2 in the kidneys of diabetic rats. Empirical evidence confirmed that SYR, through promoting autophagy pathways and alleviating oxidative stress, exerts potent renoprotective efficacy in diabetic conditions (68). In the other study performed by Rashedinia and colleagues in 2021, the diabetic Sprague-Dawley rats (STZ: 60 mg/kg; IP) received three different doses of SYR (25, 50, and 100 mg/kg) for 6 weeks. Thereupon, the renoprotective influences of SYR on anti-oxidant state and mitochondrial biogenesis in diabetic rats were examined. Whereas SYR significantly attenuated the blood glucose and TBARS, and Alkaline phosphatase (ALP) amounts in the kidney of diabetic animals, it promisingly elevated the renal GSH content and CAT and SOD actions. In addition, SYR significantly enhanced the PGC-1α and NRF-1 expression in diabetic rats compared to non-treated diabetic animals. Altogether, SYR, through targeting mitochondria in diabetic conditions, alters the anti-oxidant defense mechanism in the kidney. Thus, the findings provide a potent foundation for antidiabetic drug development by emphasizing mitochondrial health and using natural anti-oxidants (30). Toll-like receptors (TLRs) are transmembrane receptors that act in immune responses. Nowadays, concerning identifying their structural biology and signaling cascades, there is more attention to targeting them to develop medications (69). Toll-like receptor 4 (TLR4) participates in the innate immune response and then interacts with exogenous and intracellular ligands, starting complicated intracellular signaling pathways. In the same vein, TLR4 through MyD88 stimulates nuclear factor kappa-light-chain-enhancer of activated B cells (NF-κB) and Activator protein 1 (AP-1) transcription factors and thereby induces pro-inflammatory cytokines production (70). Hyperglycemia can trigger TLR-4 signaling and initiate immune responses, leading cause of inflammation and then fibrosis in the kidney cells. SYR (50 mg/kg; oral) administration after 8 weeks returned the altered biomarkers from diabetes induced by STZ (45 mg/kg; IP, a single dose) in a rat model to normal condition. SYR significantly lowered serum Cr, BUN, and protein contents in urine. Moreover, this treatment corrected the oxidant/anti-oxidant balance in the kidneys of diabetic rats. In the homogenate kidneys of diabetic rats, a decrease in the levels of biomarkers such as transforming growth factor β1 (TGF-β1), interleukin-6 (IL-6), TLR-4, and collagen after SYR treatment was observed. The results of diabetic rats who received SYR were very different compared to diabetic animals who did not receive any treatment. In general view, SYR, through preventing the TLR-4 signaling pathway, can inhibit oxidative stress, inflammation, and fibrosis in renal cells and avert diabetic nephropathy progression. The findings suggested the synthesis of promising natural antidiabetic medications targeting inflammatory pathways (71).

Finding the incidence of vision impairment and blindness in diabetic patients is crucial for informed resource allocation by nations. Nowadays, diabetic retinopathy is a global concern, especially for adults living in low and middle-income countries (72, 73). Cataracts are the leading cause of blindness worldwide, leading to decreased quality and life expectancy. Different primary factors, such as aging and oxidative stress, are associated with cataract development (74). On the other hand, diabetes can lead to an enhancement in the postoperative macular edema (PME) risk in diabetic patients undergoing cataract surgery (75). 

The effect of SYR extracted from Dendrobium on the prevention of cataract development and also aldose reductase (AR) activity in STZ-diabetic Wistar rats was evaluated. Thus, 30 mg/kg of STZ was intraperitoneally administered to rats for diabetes induction. After 30 days, each animal developed stage 2 diabetic lens opacity and was treated with SYR eye drops (2%, 50 μl, 3 times a day) for 60 consecutive days. The findings showed that protecting rats from developing induced diabetes prevented cataracts. Furthermore, SYR reduced the mRNA expression of AR and also suppressed its activity in a non-competitive and dose-dependent manner. Hence, SYR as a dual inhibitor of AR exhibited therapeutic potency for the treatment of diabetic cataracts (76).

The effect of SYR and the synergic impacts of gigantol and SYR on the prevention of cataract development, and also aldose reductase (AR) activity in STZ-diabetic Wistar rats, were evaluated. The design of these studies was according to the intraperitoneal administration of 30 mg/kg of STZ to rats. Thereupon, after 30 days, each animal suffered from diabetic lens opacity at stage 2, received SYR eye drops (2%, 50 μl, 3 times a day), and in another study received gigantol plus SYR eye drops (50 μl, 3 times a day), for 60 continuous days. Their synergistic impact was calculated using Jin’s formula and was found across a range of concentrations, with the optimal ratio being 1:1.25 gigantol to SYR. The molecular results demonstrated that Asn160 within AR is a key residue regulating the inhibitory influences of gigantol and SYR. The key findings reveal that SYR plus gigantol synergistically inhibits AR activity and expression. Additionally, the progression of cataracts in diabetic animals was inhibited. In conclusion, SYR with/or without gigantol through a disruption in the polyol pathway and blockage of AR activity contributed to a growth in anti-cataract potential in diabetic conditions (77).

Chronic wounds are a threatening health problem associated with social and economic challenges (78, 79). The wound-healing process is complicated and requires tight coordination of different actions such as migration, proliferation of cells, deposition of the matrix, remodeling, inflammation, and then angiogenesis (80). Chronic wounds deal with different comorbidities such as enhanced age, autoimmune and immunosuppression disease, microbial contamination, and diabetes mellitus (81). One of the main lower-extremity complications following diabetes is foot ulcers, which result in a significant burden for diabetic patients (82). Unfortunately, the recurrence of these ulcers is worrying in society (83). Evaluating the underlying pathological processes, incidence, management, and effectiveness of treatments in diabetic ulcer complications is necessary (84). Hence, the number of works in this area has focused on preventing and finding a promising treatment for active diabetic ulcers (82, 85). Designing the proper experimental models of diabetic foot ulcers plays an important role in drug development and, thereby, can promote the achievement of success in clinical trials (86). Since uncontrolled hyperglycemia can lead to a delay in wound healing, gangrene, and death, an attempt was made to find a natural treatment instead of accessible synthetic drugs. Topical SYR (2500, 5000 μg/L) after 14 consecutive days contributed to higher closure and epithelization of incisional wounds of diabetic male Wistar rats (Nicotinamide: 110 mg/kg + STZ: 55 mg/kg; IP, a single dose) in comparison to the diabetic control group. Moreover, SYR elevated the hydroxyproline and protein levels in diabetic rats. The effectiveness of SYR in controlling serum insulin, blood glucose levels, and dyslipidemia in diabetic animals was observed. In addition, the up-regulation of growth factors (TGF-β1, Vascular endothelial growth factor (VEGF), Alpha Smooth Muscle Actin (α-SMA), and collagen-I) and cluster of differentiation (CD) 31 and 68 expressions by SYR was reported. Fortunately, the SYR treatment improved collagen deposition and re-epithelialization, and then completed the wound closure in diabetic wound rats. SYR prescription to diabetic animals significantly prevented the oxidative stress and pro-inflammatory response (TNF-α, NF-κB, p65, IL-8, IL-2, and IL-1β synthesis) while promoting the anti-inflammatory response through IL-10 marker generation. Furthermore, SYR elevated tissue inhibitors of metalloproteinases-1 (TIMP-1) and tissue inhibitors of metalloproteinases-2 (TIMP-2) concentrations and decreased the content of matrix metalloproteinases (MMP)-2, -8, and 9 in diabetic rats. SYR alleviated the angiogenesis and macrophage polarization biomarkers, and enhanced collagen deposition, re-epithelialization, neovascularization, and skin structure in diabetic wounds. Overall, researchers stated that SYR is a promising treatment for relieving diabetic wounds due to its anti-inflammatory, anti-oxidant, angiogenic, ECM remodeling, and anti-diabetic properties (87). In this context, Padalkar and coworkers 2022 manufactured a wound care dressing for recovering diabetic wounds using SYR, *Aloe vera*, and curcumin. Hence, the fabricated hydrogel (including SYR, curcumin, and Aloe vera) was put in a sterilized polyurethane foam. The effect of polyurethane foam dressing was evaluated in diabetic albino Wistar rats (alloxan monohydrate: 120 mg/kg; IP; a single dose). The diabetic animals that used polyurethane foam dressing showed a faster rate of wound healing (14 days) than undressed animals. The fabricated wound dressing, owing to possessing curcumin, SYR, and Aloe vera, accelerated the cell proliferation and differentiation and helped to hasten healing and more wound tensile strength of diabetic wounds. In conclusion, polyurethane foam dressing fabricated by a complex of potent natural anti-oxidants can be considered an effective treatment for diabetic wound healing. This dressing improved inflammation, tissue remodeling phases, and the proliferation of diabetic wounds (88).

Sabahi and coworkers determined the protective effects of SYR on hepatic damage in diabetic rats. Correspondingly, the diabetic Sprague-Dawley rats (STZ: 60 mg/kg; IP) were treated with various doses of SYR (25, 50, and 100 mg/kg) for 6 weeks. SYR significantly lowered the rise in plasma biochemical parameters such as glucose, TG, LDL, glutamic-oxaloacetic transaminase (SGOT), and glutamic-pyruvic transaminase (SGPT) in diabetic rats. Although SYR enhanced the activity of CAT, it decreased the levels of SOD and malondialdehyde (MDA) in the liver tissue of diabetic rats. In addition, SYR significantly increased the expression of the master regulators of mitochondrial biogenesis (PGC1-α, NRF1, and NRF2). Overall, it was expressed that SYR, owing to its anti-oxidant function and effect on mitochondrial biogenesis, can prevent diabetes complications (89).

*In vitro studies*

Glycation is the synthesis of a heterogeneous group of advanced glycation end products (AGEs) that occur on long-lived biomolecules after a non-enzymatic process *in vivo* (90). The glycation process is more important in a hyperglycemic state that initiates after an interaction between reducing sugars (glucose and fructose), or their primary products of autoxidation (peroxides or hydroperoxides) with Deoxyribonucleic acid (DNA), lipids, or amino groups of proteins (91). AGEs following influx and accumulation in cells stimulate various signaling pathways, including TGF-β, c-Jun N-terminal kinase (JNK), MAPK/ERK, and NF-κB associated with inflammation, oxidative stress, and even different chronic metabolic diseases (90, 92). In this context, a study was designed to test the therapeutic effect and underlying mechanism of SYR as a potent antiglycation agent in preventing glycation-related complications in a model system. The binding constant of SYR was 3.07 ± 0.42 × 10^− 4^ M^−1^. Additionally, mass spectrometric and molecular modeling results demonstrated that Lysine (Lys) 93,261,232, and L-arginine (Arg) 194 are residues responsible for the binding of SYR in BSA. In other words, SYR has an antiglycation potential that can interact with proteins like BSA and prevent the glycation process. By blocking Lys residues, these acids declined glucose consumption and protected the synthesis of glycation end products (93). Since zinc and SYR possess anti-oxidant and metabolic properties, a modern complexing Zn (II)–SYR was fabricated. The results indicated complexing Zn (II)–SYR had more anti-oxidant, α-glucosidase inhibitory, and antiglycation activities than Zn (II) or SYR treatments. Additionally, this complex significantly prevented GSH depletion and lipid peroxidation in isolated rats’ liver and Chang liver cells. Complexing Zn (II)–SYR by elevating the hexokinase function contributed to rising glucose uptake by rats’ psoas muscle tissues and L6 myotubes. On top of that, the manufactured complex synergically elevated the phospho-Akt/pan-Akt ratio in tissues. In addition, it contributed to stronger molecular docking scores with target proteins related to diabetes than SYR. In sum, complexing Zn (II)–SYR was nontoxic for cells and has metabolic and anti-oxidant potentials that can pave the way for using it as a proper medicinal approach for diabetes side effects (94).

Hyperglycemia and oxidative stress can alter the homeostatic mechanisms such as the 5’ AMP-activated protein kinase (AMPK)/ Sirtuin 1 (SIRT1) pathway and autophagy in renal cells (95, 96). Metabolic alterations from hyperglycemia damage the autophagy process in renal cells, thereby apoptosis induction and tubular interstitial fibrosis (97). Administration of SYR (10 and 20 μM) to high glucose (30 mM)-treated rat renal epithelial cells (NRK 52E) contributed to the enhancement of the Nrf2 levels and autophagy induction ability. Moreover, SYR elevated the expression of Beclin 1, and autophagy-related proteins (Atg7, Atg5, and Atg3). These increased levels indicated increased phagophore formation and LC3 lipidation in NRK 52E cells. It can be concluded that SYR is a promising antihyperglycemic compound due to its potentiate effect on autophagy and anti-oxidant mechanisms (68).

Gigantol and SYR are derived from *Caulis Dendrobii*, a traditional Chinese plant. Both of these extracted components can prevent AR activity, and inhibit diabetic cataract development in rats. A study involved treating the human lens epithelial cells (HLECs) with a high dose of glucose (50 mmol/l glucose-DMEM) and incubating them for 72 hr with different concentrations of gigantol (0, 0.1, 0.5, 1.0, and 2 μg/ml) plus SYR (0, 0.125, 0.625, 1.25, and 2.5 μg/ml). The administration of SYR with/or without gigantol to HLECs led to the suppression of AR function alongside AR expression down-regulation. Moreover, these compounds significantly reduced the sorbitol levels in treated cells. Therefore, the prevention of AR function and polyol pathway disruption is the underlying mechanism in the anti-cataract potential of SYR and Gigantol (77).

In another study conducted by Wei *et al*., the design involved treating the human lens epithelial cells (HLECs) with a high dose of glucose (50 mmol/L glucose-DMEM) and incubating them for 72 hr with different concentrations of SYR (0.1, 0.22, and 0.4 g /l). The administration of SYR to HLECs led to the suppression of AR function alongside AR expression down-regulation in a dose-dependent and non-competitive manner. Therefore, SYR exhibited a physiological impact on glucose metabolism and cataract progression (76).

In another study, in the model of diabetic cataracts, HLECs were treated with 50 mM glucose +10% FBS in MEM. After that, 0.5 μM of SYR was added to 50 mM glucose + 10% FBS in MEM, and then the two models were incubated for 24 hr to create an SYR model of diabetic cataract HLECs. In doing so, SYR, through attenuation of swelling and degeneration of mitochondria, and nuclear chromatin condensation, improved the ultrastructure and integrity of HLECs in the presence. Atomic force microscopy (AFM) results showed an enhancement in the viscoelastic properties of HELCs after SYR treatment. Laser scanning confocal microscopy (LSCM) and Raman spectrometry analysis indicated that SYR properly modified the cytoskeletal organization and the liquidity of HELCs. This research provided new insight into the therapeutic strategy of SYR on diabetic cataracts. The underlying mechanisms were the improvement of biomechanics and cellular structure, accompanied by modification of cytoskeletal organization and lipid composition (98).

The reviewed studies demonstrate that SYR could be an antihyperglycemic agent in diabetic models, decreasing plasma glucose, elevating insulin secretion, and improving glycoprotein profiles. It also attenuates pancreatic injuries and promotes cellular regeneration. SYR improves kidney function by regulating oxidative stress and autophagy processes, prevents cataract development, and promotes wound healing through its anti-inflammatory effects in diabetic models. 

**Figure 3:**
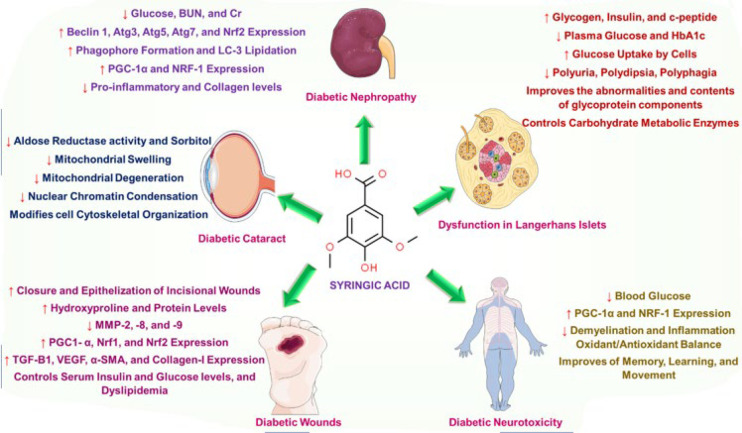
A graphic summary of the protective effect of syringic acid on diabetes complications

BUN: blood urea nitrogen; Cr: creatinine; Nrf2: nuclear factor erythroid 2–related factor 2; PGC-1α: Peroxisome proliferator-activated receptor gamma coactivator 1-alpha; NRF-1: nuclear respiratory factor 1; HbA1c: glycated hemoglobin; p-Akt: phosphorylated Akt; TGF-β1: transforming growth factor β1; Atg: autophagy; α-SMA: smooth muscle α-actin; VEGF: vascular endothelial growth factor; MMP-2: matrix metalloproteinase-2

Figure 3 is a graphic summary of the protective effect of SYR on diabetes complications.

### Effects of syringic acid on cardiovascular disease

In the current century, three risk factors, hypertension, hyperglycemia, and high BMI, are considered more worrying variables than other threats to the health of societies (99). MetS is a main risk factor in developing CVD (2, 100). The burden of mortality following cardiometabolic disorders has changed from high/low-income countries to countries with middle income. Part of the global response to non-communicable diseases (NCDs) must be devoted to reducing cardiometabolic risks via behavioral, dietary, and pharmacological interventions (101). Hence, based on the mentioned risk factors, the metabolic health concept has gained the attention of numerous researchers (99).

In the present study, we reviewed the effect of SYR on CVDs in clinical and preclinical studies. Whereas we could not find clinical research about SYR on CVDs, 10 articles in animal experimental models and 3 *in vitro* studies were evaluated. Descriptive data for the studies included are listed in Table 2.

*In vivo studies*

Sabahi *et al*. determined the protective effects of SYR on diabetic cardiomyopathy. Therefore, the diabetic Sprague-Dawley rats (STZ: 60 mg/kg; IP) received different doses of SYR (25, 50, and 100 mg/kg) for 6 weeks. Although only the high dose of SYR lowered lactate dehydrogenase (LDH) and creatine kinase-MB (CK-MB) levels, 50 and 100 mg/kg of SYR significantly decreased carbonylated protein and TBARs in STZ-diabetic animals compared to non-diabetic animals. The levels of mtDNA, GSH, and master regulators of mitochondrial biogenesis (PGC1-α, NRF1, NRF2, and transcription factor A mitochondrial (TFAM)) were altered in the hearts of diabetic animals, but SYR could not return them to normal ranges. Changes in nitric oxide (NO) levels related to mitochondrial biogenesis in diabetic animals were not observed, suggesting that the cardiac complications were not severe and had been managed through compensatory mechanisms. In sum, it was expressed that SYR, owing to its phenolic acid structure, through mitigating lipid peroxidation and protein carbonylation, exerted therapeutic effects in healing diabetic cardiomyopathy. The exact underlying mechanism involved requires more investigation (102).

It has been evidenced that oxidative stress and induced inflammation are the leading causes of diabetes complications, especially hyperlipidemia and cardiomyopathy. As SYR’s anti-oxidant and anti-inflammatory properties were covered in previous research, one experiment was planned to evaluate its effect on dyslipidemia and cardiomyopathy arising from diabetes. Thus, using an intravascular injection of STZ 45 mg/kg, type 1 diabetes was induced in female Wistar rats. Afterward, the diabetic rats received SYR (25 and 50 mg/kg; oral) for 8 weeks. The results indicated controlling hyperlipidemia and hyperglycemia, and modulation of the oxidant/anti-oxidant balance in diabetic rats by SYR. Furthermore, SYR significantly attenuated Left ventricular (LV) and cardiac hypertrophy index and histological damages in STZ-induced diabetic rats. Taken together, SYR might act through the release of inflammatory cytokines and DNA methyltransferases (DNMTs) to inhibit diabetic cardiomyopathy in the initial stages (103).

In a recent study carried out by Gao and colleagues in 2024, the model of myocardial necrosis was induced by subcutaneous injection of isoproterenol (ISO) 50 mg/kg to male albino Wistar rats on days 29 and 30 of the experiment. Thus, the protective impact of 50 mg/kg of SYR was examined in rats that underwent ISO-induced cardiotoxicity. The biochemical and *in silico* molecular docking were in agreement and confirmed that SYR significantly inhibited the HMG-CoA reductase enzyme and increased the lipoprotein lipase (LPL) enzyme action in ISO-induced myocardial necrosis rats. The *in silico* molecular docking findings further supported the modulatory impacts of these compounds on the target enzymes. The study found that the phenolic compound, SYR, could effectively modulate lipid metabolism and contribute to cardioprotection against ISO-induced myocardial necrosis by inhibiting the HMG-CoA reductase and increasing LPL enzymes (104). It has been documented that gallic acid can exert protective effects on cardiac complications like arterial hypertension, fibrosis, and cardiac hypertrophy. Hence, the cardioprotective impact of SYR, as one of the derivatives of gallic acid, on ISO-induced cardiotoxicity in mice and cells was assessed. SYR reduced heart weight, pathological damage, and fibrosis in ISO-induced cardiac necrosis in mice. The molecular findings demonstrated a significant reduction in fibrosis-related factors, including fibronectin 1 (Fn1) and collagen accumulation. Moreover, SYR down-regulated nerve growth factor receptor (Ngfr), Ereg, and Myc. Based on mechanistic findings, SYR can properly attenuate the hypertrophy and fibrosis of the heart. Overall, SYR can be considered a promising natural medicine for CVD (105). In a study, ISO 30 mg/kg for two continuous days was prescribed to male albino Wistar rats to induce myocardial infarction (MI). Afterward, the ISO-induced MI mice were treated with SYR (50 mg/kg; oral) for 7 days. cardiac marker enzymes (CK-MB, LDH, Gamma-glutamyl transferase (GGT)), and C-reactive protein (hs-CRP) serum levels were elevated in ISO-induced MI mice and were mitigated in heart tissues. SYR adjusted the oxidant/anti-oxidant balance (anti-oxidant enzymes: SOD, CAT) in ISO-induced MI mice. Post-treatment of SYR alleviated the expression of inflammatory markers (NF-κB, TNF-α, hs-CRP) in the heart of MI-mice, but recovered the body and heart weights. Whereas ISO administration led to structural damages, including myofibril fragmentation and mitochondria swelling in the heart tissues, the post-treatment of SYR prevented these ultrastructural alterations, demonstrating its potency to protect the myocardium from ISO-induced injuries. Hence, SYR is a natural agent with anti-inflammatory, anti-oxidant, and membrane-stabilizing effects and reverses the induced MI in Wistar rats (106). The pretreatment of SYR with various oral doses (12.5, 25, and 50 mg/kg) for 21 days in male Wistar albino rats in a dose-dependent manner prevented cardiotoxicity in rats that received ISO (100 mg/kg; subcutaneous injection). SYR significantly lowered CK-MB, LDH, SGPT, SGOT, protein carbonyl (PC), and inflammatory markers such as IL-6 and TNFα in the serum of ISO-induced MI rats. Moreover, the contents of anti-oxidant biomarkers were enhanced in cardiac tissue after SYR therapy. As expected, SYR relieved the erythrocyte (RBC) morphology and the infarct size in cardiotoxic rats. SYR decreased the infiltration of inflammatory cells such as neutrophils and macrophages in the myocardium and mitigated interstitial collagen deposition (cardiac fibrosis) in the myocardium tissue. In addition, SYR attenuated edema, necrosis, inflammation, and NO amounts in the heart tissue. In conclusion, SYR, owing to its intrinsic anti-oxidant and anti-lipid peroxidative properties, showed cardioprotective efficacy against ISO-induced MI. Clinical assessment is required to validate its use in humans (107).

Sammeturi *et al.* evaluated the protective effects of SYR with/without the combination of resveratrol in a cardiotoxic model of rats. For this reason, the animals were pretreated with SYR (50 mg/kg), resveratrol (50 mg/kg), or a combination of SYR (25 mg/kg) + resveratrol (25 mg/kg) + gallic acid (GA) (50 mg/kg) until day 30. The cardiotoxicity in male albino Wistar rats was induced with ISO (50 mg/kg; subcutaneous injection) on days 29 and 30. The results showed that the serum levels of CK-MB, GGT, ALT, AST, and LDH were significantly reduced by the combination pre-treatment. However, the biomarkers were remarkably raised in the homogenate heart tissue of ISO-induced cardiotoxicity rats. COMB treatment significantly enhanced the levels of anti-oxidant biomarkers such as GST, GPX, and GSH in the liver tissue of ISO-injected rats compared to the untreated ISO-injected animals. Moreover, the combination of SYR and resveratrol significantly reduced high-sensitivity hs-CRP and uric acid levels; however, it enhanced total protein content (TPC) in the serum of ISO-administered rats. It was significant that the COMB treatment restored the body and heart weights of cardiotoxic rats to normal levels. On the other hand, the levels of CK-MB, GGT, ALT, AST, LDH, GST, GPX, GSH, hs-CRP, and uric acid were not significantly different between SYR-treated ISO injected animals compared to control rats (received DMSO orally for 30 days). The findings confirmed the attenuation of cardiac biomarkers, oxidant and inflammatory indices, NF-kB, TNF-a, and TPC by COMB treatment. The study revealed that ISO resulted in mitochondrial damage, a sign of heart disease. However, COMB treatment (an anti-oxidant) reduced mitochondrial swelling through its free radical scavenging properties, likely by increasing GSH synthesis and minimizing lipid peroxidation in ISO-induced oxidative stress in rats (108).

Sammeturi *et al.* in the other study determined the cardioprotective effects of SYR with/without the combination of resveratrol in a rat model. Therefore, the animals were pretreated with SYR (50 mg/kg), resveratrol (50 mg/kg), or a combination of SYR (25 mg/kg) and resveratrol (25 mg/kg) until day 30. Cardiotoxicity was induced in male albino Wistar rats with ISO (50 mg/kg; subcutaneous injection) on days 29 and 30. The results showed that although the serum levels of CK-MB, ALP, and LDH were significantly declined by the combination of SYR and resveratrol pre-treatment, CK-MB, LDH, SOD, and CAT were remarkably raised in the homogenate heart tissue of ISO-induced cardiotoxicity rats. Moreover, this combination significantly reduced the lipid profile of ISO-administered rats, leading to decreased levels of TG, total cholesterol, VLDL-C, LDL-C, and TBARS in homogenate hearts and serum, and increased HDL-C levels. Combination therapy regulated the balance of the oxidant/anti-oxidant system in the heart tissue of MI-induced rats. The findings revealed decreased mRNA amounts of NF-kB and TNF-α in the group treated with SYR and resveratrol. Histopathological and docking findings confirmed the molecular and biochemical results. To sum up, these studies were the first to reveal that SYR and resveratrol, through attenuation of TNF-α and NF-kB signaling pathways, can exert cardioprotective effects on relieving toxicity following ISO administration (109).

In an experiment, SYR at four different doses (25, 50, 75, and 100 mg/kg) contributed to the recovery of the rat model of myocardial ischemia-reperfusion injury (MIRI). The molecular findings revealed a significant enhancement in the Bcl-2, phosphorylated Akt (p-Akt), phosphoinositide 3-kinase (PI3K), p-GSK-3β, and mitochondrial cytochrome c levels in the Sprague-Dawley rat model of MIRI by SYR. Nevertheless, SYR pretreatment alleviated the caspase-3, -9, and BCL2-associated X (Bax) expressions. The complementary results confirmed the protective effects of SYR on the activity of myocardial systolic (left ventricular ejection fraction (LVEF) and left ventricular fractional shortening (LVFS)). Furthermore, SYR resulted in a decrease in CK-MB and LDH levels in serum, as well as a reduction in the index of apoptosis in the MIRI model. Conclusively, SYR, through suppression of mitochondria-induced apoptosis modulated by the PI3K/Akt/GSK-3β signaling pathway, relieved myocardial ischemia/reperfusion injury. The drawbacks of this study were the lack of evaluation of the other signaling pathways, such as MAPK, and the investigation of the anti-oxidant anti-inflammatory involvement of SYR in its cardioprotective effects (42).

In another study, the researchers aimed to evaluate the influences of SYR on hypertension induced by 40 mg/kg of N^ω^-nitro-l-arginine methyl ester (l-NAME; oral; 4 weeks). It was observed that SYR (25, 50, and 100 mg/kg; oral) prescription after 4 consecutive weeks returned altered levels of systolic blood pressure, plasma nitric oxide metabolites (NOx), biomarkers involved in kidney and liver function, vitamin C, and vitamin E to normal values in blood and tissues of l-NAME-induced hypertensive rats compared to healthy rats. Consequently, it modulated the oxidant and anti-oxidant markers in the tissues and blood of hypertensive rats relative to the control group. It is worth noting that 50 mg/kg of SYR provided the best protection. The histopathological results confirmed biochemical findings. To sum up, SYR, owing to its anti-oxidant properties and preserves the NO bioavailability, contributes to cardiovascular protective effects (110).

*In vitro studies*

In recent years, much attention has been paid to targeting inflammation and oxidative stress in treating myocardial diseases. Shahzad and colleagues aimed to evaluate the therapeutic effects of SYR on peripheral blood mononuclear cells (PBMCs) in patients with recent heart attacks. Hence, the isolated PBMCs from MI patients and healthy individuals were cultured with SYR (5, 25, 50, 100 5 μM) for 24 hr. The obvious findings demonstrated SYR’s potential in alleviating inflammatory biomarkers such as TNF-α, IL-6, and NO as well as anti-oxidant markers (ROS, lipid, and protein oxidation) in MI patients compared to healthy individuals. Moreover, SYR exhibited reasonable protective effects on biomolecular structure in treated PBMCs. The data derived from molecular docking analysis indicated that SYR had a considerable binding affinity for IL-6 and TNF-α. In conclusion, SYR exerted anti-oxidant and anti-inflammatory properties, likely by direct interactions with main enzymes and signaling pathway proteins, and protected the functional integrity and structure of cellular biomolecules in MI (111).

In another study, a model of human pulmonary artery endothelial cells (HPAEC) injury using lipopolysaccharides (LPS) was developed. Then, the effect of SYR on vascular resistance was evaluated. The findings showed a significant improvement in vascular injury following SYR treatment. The vascular cell adhesion molecule 1 (VCAM-1), intercellular adhesion molecule-1 (ICAM-1), monocyte chemoattractant protein-1 (MCP-1), endothelin 1 (ET-1), e-selectin, inducible nitric oxide synthase (iNOS), TNF-α, IL-6, and IL-1β levels were lowered after SYR treatment, while the levels of NO and nuc-p65 were enhanced. Moreover, SYR significantly up-regulated the p-AMPK, IκBα, and cyto-p65 expressions. Finally, the AMPK/NF-κB signaling pathway was introduced as the involved mechanism in the protective effects of SYR on vascular resistance. The results demonstrated that SYR possesses remarkable therapeutic effectiveness in managing vascular inflammation via different biochemical pathways and cellular mechanisms. SYR has shown promise in preventing vascular inflammation in clinical settings, especially in HPAEC subjected to LPS-induced injury (112).

While it had been generally agreed that SYR can decrease collagen-induced platelet aggregation, there was less research on the effect of this phytochemical on clot formation (thrombosis) and platelet activation. For this reason, an experiment was designed to evaluate its impact on platelet activity. It was observed that SYR suppressed the function of procoagulant protease, prevented clot formation, and even destroyed blood clots. Consequently, SYR significantly down-regulated the expression of positive regulators of thrombosis, including density-enhanced phosphatase-1 (DEP-1), αIIbβ3, and protein tyrosine phosphatase-1B (PTP1B). In addition, SYR reduced the phosphorylation of kinases such as protein kinase B (AKT), PI3K, extracellular signal-regulated kinase (ERK), c-Jun N-terminal kinase (JNK), and P38, as well as integrin αIIbβ3 involved in epinephrine/collagen-stimulated platelets in mice blood and *in vitro*. Furthermore, SYR prevented the secretion of granule constituents, clot retraction, and carotid vascular occlusion that had been induced by FeCl3 administration. In summary, SYR via DEP-1/PTP1B/αIIbβ3/kinases prevented the formation of fibrin clots, platelet stimulation, and coagulant factors and alleviated thromboembolism and thrombosis. SYR indicated a multifaceted approach to prevent thrombosis and platelet function, making it a promising candidate for therapeutic interventions in cardiovascular health. Its mechanisms were involved in delaying clot formation and platelet function by interrupting the interaction between thrombin and fibrinogen, reducing the activity of procoagulant proteases, prolonging the extrinsic coagulation cascade, decreasing granule secretion, and down-regulating the signaling of DEP-1/PTP-1B/αIIbβ3/kinases (113).

Studies show that SYR can protect against myocardial infarction by reducing cardiac injury markers and mitigating oxidative stress. Its anti-oxidant and anti-inflammatory properties manage lipid peroxidation and protein carbonylation, alleviating early-stage cardiac complications. Combination therapy reduces cardiotoxicity markers and improves lipid profiles. SYR’s potential for clinical applications in managing myocardial diseases is supported.

**Figure 4:**
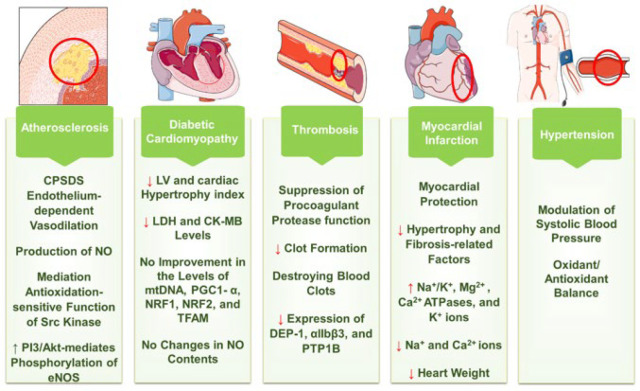
A summary of the protective effect of syringic acid on cardiovascular complications

Figure 4 displays a summary of the protective effect of SYR on CVDs.

### Effects of syringic acid on dyslipidemia

Dyslipidemia manifests with high levels of triglycerides (TG) and cholesterol in serum (114). In other words, in dyslipidemia, the lipid profile is considered abnormal if low-density lipoprotein cholesterol (LDL-C) is ≥130 mg/dl, total cholesterol is ≥240 mg/dl, or high-density lipoprotein cholesterol (HDL-C) is <40 (22). This impairment is a major risk factor in CVD development, like atherosclerosis (115, 116). Many risk factors, such as age, gender, familial history, education, food habits, drinking, smoking, diabetes, hypertension, CVDs, and stroke, could be accompanied by dyslipidemia (117). In sum, since the rise in serum levels of lipid profile is interpreted as hyperlipidemia and hyperlipidemia is a subset of MetS disorders, improving lipid profile reduces the incidence of MetS.

Hence, we reviewed the articles about the effect of SYR on lipid profiles in clinical and preclinical research. Although we could not find clinical research about SYR’s effect on hyperlipidemia, five articles in animal experimental models and two *in vitro* studies were assessed. Table 3 presents the results from the descriptive data about the SYR effect on lipid profiles in preclinical research.

*In vivo studies*

Pulmonary inflammation, which can result from conditions like asthma, chronic obstructive pulmonary disease (COPD), and influenza, may be alleviated by SYR, a bioactive compound with therapeutic properties. A study conducted on male Wistar rats revealed that a dosage of SYR at 50 mg/kg significantly reversed dyslipidemia induced by an HFD and improved oxidative stress markers associated with this diet. Additionally, SYR was found to lower levels of inflammatory cytokines, albumin, LDH, and globulin. It also exhibited anti-oxidative and anti-inflammatory effects by inhibiting the activation of pro-inflammatory cytokines. These findings indicate that SYR may provide protective effects against pulmonary injuries caused by a high-fat diet, potentially through the modulation of inflammatory responses and oxidative stress. Further research is necessary to understand the precise mechanisms behind these effects and to explore SYR’s potential for use in future therapeutic applications (118). 

Zaini *et al.* aimed to evaluate the antihyperlipidemic action of the 9.45 mg/kg eutectic mixture of SYR and fenofibric acid (lipid-lowering drug) in male Wistar rats. SYR was used as a coformer to improve the dissolution rate, solubility, and lipid-lowering potential of fenofibric acid. After 15 days, the blood cholesterol levels in rats were measured. The biochemical results demonstrated a significant reduction in total cholesterol levels in groups that received eutectic mixtures compared to rats treated with fenofibric acid. In sum, forming a eutectic mixture of fenofibric acid with SYR remarkably improved both its solubility and dissolution rate, leading to increased antihyperlipidemic function. These results highlighted the potential for using these eutectic mixtures as a strategic approach in pharmaceutical formulations to improve the bioavailability of poorly soluble drugs (119).

Regarding the promotion of the bioavailability, solubility, and antilipidemic effects of SYR, a self-micro emulsifying drug delivery system (SMEDDS) was manufactured. The formulation exhibited prolonged half-life (t1/2), time to maximum plasma concentration (Tmax), and Mean Residence Time (MRT) compared to pure SYR after prescription to high-fat diet (HFD) mice. Moreover, fabricated SYR-SMEDDS increased liver accumulation and bioavailability, and delayed the elimination of SYR in hyperlipidemic mice. Hence, SYR-SMEDDS significantly decreased serum lipid profiles in HFD mice and relieved liver steatosis. The findings suggest that the SYR-SMEDDS nanosystem not only elevated the oral bioavailability of SYR but also promoted hypolipidemic influences via molecular mechanisms involving improved solubility, controlled release, and elevated cellular uptake. This approach represented a promising insight for developing efficient treatments for hyperlipidemia and likely metabolic disorders (120).

The protective effects of SYR (50 mg/kg, oral) on hepatotoxicity from acetaminophen (APAP; 750 mg/kg) were examined in Wistar albino rats. The administration of SYR resulted in decreased levels of TG, free fatty acids, phospholipids, and total cholesterol. SYR significantly elevated anti-oxidant biomarkers such as CAT and SOD, glutathione (GSH), GPx, vitamin E, and vitamin C in rats after 8 days. Additionally, SYR attenuated the content of lipid peroxidative biomarkers, including lipid hydroperoxides and TBARS in animals (121). However, for the second time, SYR was prescribed with three different doses (25, 50, and 100 mg/kg; oral) to APAP rats. SYR decreased the LDL-C levels and very low-density lipoprotein cholesterol (VLDL-C) while increasing HDL-C phospholipids in rat serum. Moreover, SYR attenuated TG, free fatty acids, phospholipids, and total cholesterol levels in plasma, kidney, and liver tissues. At last, SYR was introduced as a promising antihyperlipidemic agent. In summary, SYR indicated remarkable hepatoprotective and antihyperlipidemic impacts via several mechanisms, including decreasing hepatic enzyme amounts indicative of liver injuries, improving lipid profiles disrupted following APAP toxicity, and exhibiting anti-oxidant effects. These findings proposed their potential therapeutic application in controlling drug-induced liver damage and dyslipidemia (121).

*In vitro studies*

John and Arockiasamy assessed the anti-adipogenic effect of SYR on 3T3-L1 murine preadipocytes. It was hypothesized that SYR may have anti-adipogenic effects because of inhibition of differentiation and control of lipid accumulation in preadipocytes. Therefore, 3T3-L1 preadipocytes were incubated with SYR (100–1000 µmol/ml) for two and ten days, and then LDH release, MTT assays, and Oil Red O staining were performed. The Oil Red O staining results indicate that the concentration of SYR inhibited the differentiation of 3T3-L1 preadipocytes into mature adipocytes in a dose-dependent manner over ten days. This suppression was due to the prevention of mitotic clonal expansion in the initial stages of differentiation. Moreover, SYR enhanced lipolysis (glycerol release) and decreased lipogenesis (TG synthesis) in fat cells. Besides, SYR significantly lowered ROS synthesis in matured adipocytes. It was demonstrated that SYR has anti-adipogenic and anti-oxidant effects in 3T3-L1 adipocytes (122).

John and Arockiasamy, in another similar study, determined the anti-adipogenic and anti-oxidant potential of SYR on 3T3-L1 murine preadipocytes. Therefore, the 3T3-L1 preadipocytes were incubated with SYR (100–1000 µmol/ml) for two and ten days. The results indicated SYR potentially regulated the oxidant/anti-oxidant balance, which significantly lowered NADPH oxidase 4 (NOX4) expression and ROS synthesis, and thereby recovered the amounts of GSH, CAT, and SOD in matured adipocytes. It was demonstrated that SYR has anti-radical scavenging and anti-oxidant effects in 3T3-L1 adipocytes. In conclusion, SYR can be considered an effective natural candidate for treating oxidative stress arising from obesity. This can attenuate inflammation and MetS (123).

The reviewed studies indicated that SYR can decrease cholesterol levels, including LDL and VLDL-C, via increasing lipolysis and decreasing lipogenesis in adipocytes. SYR treatment also balanced the oxidant/anti-oxidant system, enhanced Vitamin E and Vitamin C, and relieved liver steatosis. *In vitro* research showed that SYR suppresses the differentiation of preadipocytes to mature adipocytes, increases lipolysis, and possesses anti-oxidant influences. SYR not only decreases harmful lipids but also promotes HDL-C, suggesting its potency as a probable natural antihyperlipidemic agent. 

**Figure 5:**
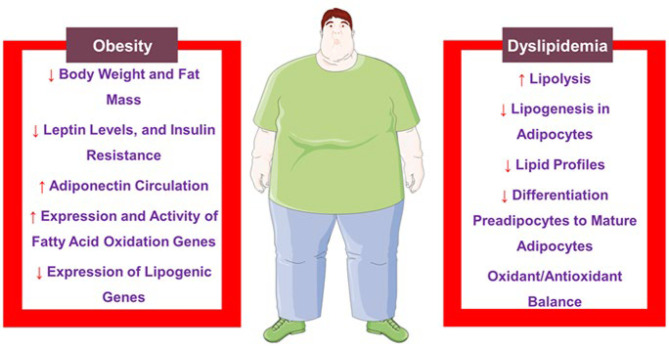
A summary of the hypolipidemic and anti-obesity effects of syringic acid

Figure 5 displays a summary of the hypolipidemic effects of SYR.

### Effects of syringic acid on obesity

In the latest guidelines, obesity is defined as a waist circumference ≥40 inches (102 cm) in men, and ≥35 inches (88 cm) in women (22, 124). Obesity is associated with severe health outcomes during a person’s lifetime (125, 126). Nowadays, due to the alteration of lifestyles, the prevalence of overweight is growing, resulting in rising complications related to MetS, especially dyslipidemia, hypertension, and diabetes, thereby increasing CVDs (124). 

It has been evidenced that healthy nutrition plays an important role in preventing or returning the overweight to a normal state (125, 127). Therefore, in this section, we reviewed the research evaluating the effect of SYR as a phytochemical on obesity in clinical and preclinical research. We could not find clinical research about inhibitory potential of SYR on obesity. On the other hand, four articles in animal experimental models and one *in vitro* study were assessed. Descriptive data for the studies included are listed in Table 4.

*In vivo studies*

Recently, one research was planned by Khatun *et al*. This research tended to find the anti-obesity effect of SYR in HFD mice. Thus, the mice were continuously fed an HFD for 12 weeks. After 12 weeks, an enhancement in Body mass index (BMI), body weight, and metabolic biomarkers was observed. For evaluating the anti-obesity effect of SYR in overfeeding mice, 50 mg/kg SYR was prescribed to mice for 4 weeks. Accordingly, SYR declined lipid biomarkers related to obesity in overfat mice. Collectively, SYR may be a reasonable therapeutic compound for overweight individuals, however, further clinical research is required (128).

The Zhang study investigated the SYR effects on nonalcoholic fatty liver disease (NAFLD) using a rat model fed an HFD. The rats were divided into four groups: a control group, an HFD group, an SYR-administered HFD group, and a positive control group receiving SYR on a normal diet. SYR (20 mg/kg) was prescribed orally for eight weeks, during which it effectively controlled lipid profiles, decreased serum levels of AST and ALT, suppressed pro-inflammatory cytokines, and attenuated histopathological and immunohistochemical alterations. Furthermore, SYR reversed oxidative stress by 82% and replenished anti-oxidant functions. In the SYR-treated rats, the gene expressions of Nrf2/heme oxygenase 1 (Nrf2/HO-1) were enhanced. The beneficial effects of SYR on NAFLD were significant, primarily via the reversal of oxidative stress and inflammation (129). 

In another experiment, the C57BL/6J male mice received HFD with/ without SYR (0.05%, wt/wt) for 16 weeks, and the anti-obesity efficacy of SYR was estimated. The findings indicated a reduction in visceral fat mass, body weight, leptin levels, lipid content of the liver, and insulin resistance after SYR treatment. However, SYR enhanced adiponectin circulation. Although SYR increased the expression of fatty acid oxidation genes, such as Cpt1, Cpt2, Acsl, and Pparα in the liver of HFD mice, it also brought about a decrease in the expression of lipogenic genes, including Srebp-1c, Srebp-2, Cidea, Fasn, Pparγ, and Hmgcr. Additionally, SYR significantly enhanced the activity of fatty acid oxidation enzymes. It alleviated lipogenic enzyme activities in the liver tissue of treated HFD mice compared to HFD mice that had not been treated with SYR. Overall, SYR, through adjusting the genes involved in inflammation and lipid metabolism, exerted anti-inflammatory, anti-obesity, and anti-steatotic activities. Hence, it was suggested that SYR could be assumed a promising natural therapy for obesity (39).

Menopause is a natural and physiological phase in an older woman’s life (130, 131). The old women undergo estrogen deficiency and experience common vasomotor symptoms including hot flashes and night sweats, muscle, and joint pain, sleep disturbances, and vaginal dryness (130). Menopause is associated with cardiometabolic alterations and increases the aging impact on the risk of CVD progression. The menopausal transition is manifested in an increment in body fat mass, hyperlipidemia, insulin resistance, and endothelial dysfunction (132). One solution for decreasing postmenopausal complications is using beneficial dietary supplements. For this reason, the ovariectomized (OVX) mice received 100 mg/kg of SYR in their diet for 12 weeks. The HFD was used to induce obesity in OVX mice. Dietary SYR did not influence food consumption and body weight in control OVX mice, but in the OVX mice on HFD, it decreased body fat mass (subcutaneous, visceral, and total fat). Furthermore, SYR significantly lowered the serum levels of TG. In summary, it can modulate lipid metabolism in OVX mice. Hence, it is a good idea to use SYR in preventing postmenopausal obesity (133).

*In vitro studies*

Chowdhury and colleagues in 2020 aimed to evaluate the inhibitory potential of SYR on obesity using molecular docking analysis. The molecular docking of SYR with vital proteins related to obesity was determined. Molecular docking findings revealed higher inhibitory scores of SYR (-5.491 kcal/mol) than Orlistat (-3.881 kcal/mol; an anti-obesity drug; positive control) against obesity. In conclusion, performing more clinical and pre-clinical research was strongly proposed to cover the therapeutic effects of SYR (134).

Research showed that daily consumption of SYR can elevate fatty acid-oxidizing enzyme activity and mitigate lipogenic enzymes, decreasing lipid biomarkers involved in obesity. This reduces body fat mass and weight, lowers insulin resistance, and elevates adiponectin circulation. *In vitro* studies indicated SYR has greater inhibitory scores than Orlistat against obesity, demonstrating its probable potential as a natural therapy for obesity. 

Figure 5 shows a summary of the anti-obesity benefits of SYR.

### Drug interactions and possible side effects of syringic acid

SYR has demonstrated a good safety profile in preclinical studies, indicating few side effects at therapeutic doses. However, potential drug interactions and side effects should be considered based on existing mechanistic and toxicity data.

*Safety profile*

In a subacute toxicity study, rats treated with SYR at 1000 mg/kg/day orally for 14 days showed no significant adverse effects on body weight, food intake, hematological parameters, or organ function. Histopathological analysis revealed no damage to their internal organs. There is a lack of subchronic and chronic toxicity studies; however, subacute data suggest that the effects may be reversible after discontinuation (135).

*Potential drug interactions*

The pharmacological activity of SYR involves multiple pathways, indicating potential interactions with the following:

The antinociceptive effects of SYR were significantly reversed by naloxone, an opioid antagonist, indicating an interaction with opioid receptors. Concurrent use of opioids such as morphine may alter their efficacy (136). Moreover, Pre-treatment with yohimbine, an α2-adrenoceptor antagonist, reduced SYR’s analgesic effects, indicating a relationship with adrenergic signaling (136). Mecamylamine, a nicotinic acetylcholine receptor antagonist, inhibited the antinociceptive activity of SYR, suggesting that there are interactions with nicotinic pathways (136). 

*Side effects*

SYR is generally safe, with few reported cases of significant toxicity observed in animals. However, its safety and side effects in humans are not yet established. While SYR shows no major side effects at therapeutic doses in animal models, further clinical studies are needed to confirm its safety in humans (37, 136, 137). SYR’s safety and drug interactions in humans remain unknown; effective doses in rodents are lower than high doses. While SYR appears safe in preclinical models, caution is advised when combining it with opioid, adrenergic, or cholinergic drugs.

### Future prospective

SYR is a potential therapeutic agent for diabetes and MetS, and it has shown promising results in preclinical studies. However, clinical trials are necessary to confirm its efficacy, safety, and mechanisms of action. This involves designing randomized controlled trials, determining optimal dosing and safety, evaluating long-term effects, and investigating the effects in humans. Until these studies are conducted, the use of SYR in clinical practice lacks direct evidence from human studies.

## Conclusion

Based on this review, SYR demonstrated protective effects on MetS clusters, including diabetes, CVDs, hyperlipidemia, and obesity. In animal models, SYR could reduce blood glucose levels, improve insulin secretion, protect pancreatic β-cells, modulate carbohydrate metabolism enzymes, and decrease oxidative stress. Moreover, it could mitigate cardiac damage markers, mitigate oxidative stress, improve lipid profiles, and alleviate cardiac hypertrophy and fibrosis. Furthermore, SYR helps to manage obesity by lowering body weight and fat mass, attenuating leptin levels, enhancing adiponectin circulation, improving insulin resistance, and modulating genes involved in lipid metabolism. SYR affected different pathways involved in MetS, such as pancreatic β-cell protection, improved insulin sensitivity, regulation of enzymes, inflammation decrement, mitochondrial function increment, and autophagy regulation. On the other hand, synergistic effects between SYR and other compounds like resveratrol were observed. Since there is a gap in research on its effects on MetS in humans, further research is suggested to assess SYR’s efficacy and safety in clinical settings to confirm preclinical findings. The heterogeneity of reviewed studies made it challenging to draw definitive conclusions. However, the current study provides insight into further assessment of SYR as an adjunct therapeutic candidate for MetS. We suggest future studies focus on randomized controlled trials, optimal dosages, and exploring SYR’s mechanisms of action.

**Table 1:** Protective effects of syringic acid on diabetes mellitus

**Source**	**Dose/Concentration**	**Study model**	**Results**	**Ref.**
**SYR**	10 and 25 mg/kg day; oral/ last week	Male NMRI mice treated with 3 mg/kg; oral/ for 30 days	↓ Liver enzyme levels, FBS, GTT, NO, TNF-α, and TBARS ↑ Total thiol levels, CAT, SOD, GPx, and caspase-3 expression	(58)
**SYR**	25, 50, and 100 mg/kg day; oral/ for 30 days	Albino Wistar rats treated with Alloxan 150 mg/kg; IP/ a single dose	↑ Glycogen, insulin, and hemoglobin levels ↓ Plasma glucose and HbA1c ↓ Pancreatic damage Controlling the function of carbohydrate metabolic enzymes	(59)
**SYR**	50 mg/kg day; oral/ for 30 days	Albino Wistar rats treated with Alloxan 150 mg/kg; IP/ a single dose	↓ Plasma glucose levels ↑ Amounts of plasma insulin and C-peptide Normalizes the content of tissue and plasma glycoproteins Improves the abnormalities of glycoprotein components	(36)
**SYR**	50 mg/kg/day; oral/ for 60 days	Sprague-Dawley rats treated with STZ 40 mg/kg, IP/as a single dose	↓ Blood glucose and HbA1c levels Improved insulin levels ↓ Glucose-6 phosphatase and fructose 1,6-bisphosphatase activities ↑ Function of hexokinase and pyruvate kinase Balance the oxidant/antioxidant system	(60)
**SYR**	25 mg/kg, and 50 mg/kg; oral/ from 8^th^ to 18^th^ postnatal weeks	Wistar rat neonates treated with STZ, split 110 mg/kg, IP/ on 2 and 3 postnatal days	↓ Hyperglycemia, polyuria, polydipsia, polyphagia, and HbA1c levels ↓ Indices of cardiac hypertrophy and relative organ weight Balance the oxidant/antioxidant system	(37)
**Zinc (II)-SYR**	Zinc (15 mg/kg) or/ and SYR (45 mg/kg); oral/ for 4 weeks	Male Sprague Dawley rats treated with 10% fructose plus 40 mg/kg STZ; IP/ a single dose	More antidiabetic effects than each Zn (II) or SYR ↓ Hyperphagia and polydipsia Moderating the glycogen synthesis in tissues, Akt phosphorylation, sensitivity and secretion of insulin, and the activity of muscle hexokinase Balanced the oxidant/antioxidant system	(61)
**SYR**	25, 50, and 100 mg/kg; oral/ for 6 weeks	Sprague-Dawley rats treated with STZ 60 mg/kg; IP/ a single dose	Relieved memory, learning, and movement ↑ Expression of PGC-1α and NRF-1 in rat brains ↓ Demyelination and inflammation in sciatic nerves Balanced oxidant/antioxidant system	(64)
**SYR**	25 and 50 mg/kg; oral/ for 4 weeks after 4 weeks of diabetes induction	Male Sprague–Dawley rats treated with STZ 55 mg/kg; IP/ a single dose	↑ Expression of light chain (LC) 3-IIB in the kidney ↓ Levels of serum creatinine, urine creatinine, and urea Improved the expression of autophagy-related proteins (Atg3, Atg5, and Atg7), and Nrf2 in the kidney	(68)
**SYR**	25, 50, and 100 mg/kg; oral/ for 6 weeks	Sprague-Dawley rats treated with STZ 60 mg/kg; IP/ a single dose	↓ Blood glucose TBARS, and ALP amounts in the kidney ↑ PGC-1α and NRF-1 expression Balanced the oxidant/antioxidant system	(30)
**SYR**	50 mg/kg; oral/ for 8 weeks	Albino Sprague Dawley rats treated with STZ 45 mg/kg; IP/ a single dose	↓ Serum creatinine, BUN, and protein contents in urine ↓ Levels of TGF-β1, IL-6, TLR-4, and collagen Balanced the oxidant/antioxidant system	(71)
**SYR**	50 μl of eye drops, 3 times a day/ for 60 days	Wistar rats treated with STZ 30 mg/kg; IP/ a single dose	↓ AR activity and expression Inhibition of the progression of cataracts	(76)
**Gigantol Plus SYR**	50 μl of eye drops, 3 times a day/ for 60 days	Wistar rats treated with STZ 30 mg/kg; IP/ a single dose	↓ AR activity and expression Inhibition of the progression of cataracts	(77)
**SYR**	2500 and 5000 μg/L; topical/ for 14 days	Male Wistar rats treated with Nicotinamide 110 mg/kg + STZ 55 mg/kg; IP/ a single dose	Higher closure and epithelization of incisional wounds Improved collagen deposition ↑ Hydroxyproline and protein levels Controlling serum insulin, glucose levels, and dyslipidemia ↑ Growth factors (TGF-Β1, VEGF, α-SMA, and collagen-I) and CD 31 and 68 expressions ↓ Oxidative stress and pro-inflammatory response (TNF-α, NF-κB p65, IL-8, IL-2, and IL-1Β synthesize) ↑ Anti-inflammatory response through IL-10 marker generation ↑ TIMP-1 & TIMP-2 concentrations ↓ Content of matrix metalloproteinases (MMP-2, -8, AND -9)	(87)
**Polyurethane foam dressing **	For 14 days	Albino Wistar rats treated with Alloxan 120 mg/kg; IP/ a single dose	Acceleration of cell proliferation and differentiation Fast healing	(88)
**SYR**	25, 50, and 100 mg/kg; oral/ for 6 weeks	Sprague-Dawley rats treated with STZ 60 mg/kg; IP/ a single dose	↓ Glucose, TG, LDL, SGOT, and SGPT Balanced the oxidant/antioxidant system ↑ Expression of master regulators of mitochondrial biogenesis (PGC1- α, Nrf1, and Nrf2	(89)
**SYR**	50, 100, 150 μg/ml for 21 days	BSA glucose model BSA 20 mg/ml + glucose 500 mM	Binding constant was 3.07 ± 0.42 × 10^− 4^ M^− 1^ Lys 93,261,232, and Arg 194 are residues responsible for the binding of SYR	(93)
**Zn (II)–SYR**	-	Isolated rats' liver and Chang liver cells rats' psoas muscle tissues and L6 myotubes treated with glucose 500 mM/ for 24 hr	α-glucosidase inhibitory, and antiglycation activities Balanced the oxidant/antioxidant system ↑ Hexokinase function and glucose uptake Stronger molecular docking scores with target proteins	(94)
**SYR**	10 and 20 μM/ for 24 hr	NRK 52E cells treated with glucose 30 mM/ for 24 hr	↑ Nrf2 levels and autophagy induction ability ↑ Expression of Beclin 1, and autophagy-related proteins (Atg7, Atg5, and Atg3) ↑ Phagophore formation and LC3 lipidation	(68)
**SYR**	1, 2, and 4 mg/ml/ for 24 hr	HLEC cells treated with glucose 50 mmol/l glucose/ for 72 hr	Suppression of AR function AR expression down-regulation ↓ Sorbitol levels	(76)
**Gigantol plus SYR**	Gigantol (0, 0.1, 0.5, 1.0, and 2 μg/ml) plus SYR (0, 0.125, 0.625, 1.25, and 2.5 μg/ml)/ for 72 hr	HLEC cells treated with glucose 50 mmol/l glucose/ for 72 hr	Suppression of AR function AR expression down-regulation ↓ Sorbitol levels	(77)
**SYR**	0.5 μM/ for 24 hr	HLEC cells treated with 50 mM glucose +10% FBS/ for 24 hr	↓ Swelling and degeneration of mitochondria and nuclear chromatin condensation Improved the ultrastructure and integrity of HLEC ↑ Viscoelastic properties of HLEC Modified the cytoskeletal organization and liquidity of HLEC	(98)

α-SMA: smooth muscle α-actin; AR: aldose reductase; ATG: autophagy related; BUN: blood urea nitrogen; CAT: catalase; DPP IV: dipeptidyl peptidase IV; HbA1c: glycated hemoglobin, HLECs: human lens epithelial cells; IL-6: interleukin-6; IP: intraperitoneal; LDL: low-density lipoprotein cholesterol; MetS: metabolic syndrome; MMP-2: matrix metalloproteinase-2; NRK 52E: rat renal epithelial cells; NRF-1: nuclear respiratory factor 1; Nrf2: nuclear factor erythroid 2–related factor 2; Polyurethane foam dressing: Fabricated hydrogel (including SYR, curcumin, and Aloe vera) was put in a sterilized polyurethane foam; PGC-1α: Peroxisome proliferator-activated receptor gamma coactivator 1-alpha; SGOT: glutamic-oxaloacetic transaminase; SGPT: glutamic-pyruvic transaminase, SOD: superoxide dismutase; STZ: streptozotocin; SYR: syringic acid; TG: triglycerides; TGFβ1: transforming growth factor β1; TIMP 1,2: tissue inhibitors of metalloproteinase 1 and 2; TLR4: toll-like receptor 4; TNF-α: tumor necrosis factor alpha; VEGF: vascular endothelial growth factor

**Table 2:** Protective effects of effects of syringic acid on cardiovascular disease

**Source**	**Dose/Concentration**	**Study model**	**Results**	**Ref.**
**SYR**	25, 50, and 100 mg/kg; oral/ for 6 weeks	Sprague-Dawley rats treated with STZ 60 mg/kg, IP/as a single dose	↓ LDH, and CK-MB levels ↓ Carbonylated protein and TBARs No improvement in the levels of mtDNA, GSH, and mitochondrial biogenesis indices (PGC1-α, NRF1, NRF2, and TFAM) No changes in NO contents	(102)
**SYR**	25 and 50 mg/kg; oral/ for 8 weeks	Female Wistar rats treated with STZ 45 mg/kg; IV/ a single dose	Controlling hyperlipidemia and hyperglycemia ↓ LV and cardiac hypertrophy index and histological damages Balanced the oxidant/antioxidant system	(103)
**SYR**	50 mg/kg; oral/ for 30 days	Male Albino Wistar rats treated with ISO 50 mg/kg; SC/on days 29 and 30	Consistent biochemical and *in silico* molecular docking results Inhibited HMG-CoA reductase enzyme ↑ LPL enzyme action	(104)
**SYR**	-	Mice and cells treated with ISO	↓ Heart weight, pathological damage ↓ Fibrosis-related factors like Fn1 and collagen accumulation ↓ Ngfr, Ereg, and Myc ↓ Nppb and Fn1 up-regulations and the size of cells ↓ Hypertrophy and fibrosis of the heart	(105)
**SYR**	50 mg/kg; oral/ for 7 days	Male albino Wistar rats treated with ISO 30 mg/kg; SC /on days 1 and 2	↓ Expression of NF-kB and TNF-α Recovered the body and heart weights Rehabilitated the myocardial complications Balanced the oxidant/antioxidant system	(106)
**SYR**	12.5, 25, and 50 mg/kg; oral/ for 21 days	Male Wistar Albino rats treated with ISO 100 mg/kg; SC / on days 20 and 21	↓ CK-MB, LDH, ALT, AST, PC, and inflammatory markers such as IL-6 and TNFα in the serum Relieved RBC morphology and the infarct size Balanced the oxidant/antioxidant system Consistent biochemical and histological findings	(107)
**SYR**	SYR (50 mg/kg), resveratrol (50 mg/kg), or a combination of SYR (25 mg/kg) + resveratrol (25 mg/kg) + gallic acid (GA) (50 mg/kg); oral/ for 30 days	Male Albino Wistar rats treated with ISO 50 mg/kg; SC/ on days 29 and 30	↓ Serum levels of CK-MB, GGT, ALT, AST, and LDH. ↑ CK-MB, GGT, ALT, AST, and LDH in heart tissue ↑ GST, GPX, and GSH in the liver tissue Balanced the oxidant/antioxidant system Restored the body and heart weights ↓ hs-CRP and uric acid levels ↑ TPC in serums	(108)
**SYR**	SYR (50 mg/kg), resveratrol (50 mg/kg), or a combination of SYR (25 mg/kg) and resveratrol (25 mg/kg); oral/ 30 days	Male Albino Wistar rats treated with ISO 50 mg/kg; SC/ on days 29 and 30	↓ Serum levels of CK-MB, ALP, and LDH ↑ CK-MB, LDH, SOD, and CAT in heart tissue ↓ Lipid profile in homogenate hearts and TG, total cholesterol, VLDL-C, LDL-C, and TBARS in serum ↑ HDL-C amounts Balanced the oxidant/antioxidant system Recovered m-RNA amounts of NF-kB and TNF-α Consistent Histopathological, Docking, molecular and biochemical findings	(109)
**SYR**	25, 50, 75, and 100 mg/kg; oral/ for 3 days	Male Sprague-Dawley rat model with myocardial ischemia-reperfusion injury	↑ Bcl-2, p-Akt, p-PI3K, p-GSK-3β, and mitochondria cytochrome c levels ↓ Caspase-3, -9, and Bax expressions Protection of myocardial systolic (LVEF and LVFS) ↓ CK-MB and LDH levels in serum ↓ Index of apoptosis Suppression of mitochondria-induced apoptosis Modulating by the PI3K/Akt/GSK-3β pathway Relieved myocardial ischemia/reperfusion injury	(42)
**SYR**	25, 50, and 100 mg/kg; oral/ for 4 weeks	Male Albino Wistar rats treated with l-NAME; oral/ for 4 weeks	Returned altered levels of systolic blood pressure, plasma NOx, kidney and liver function biomarkers, vitamin C, and vitamin E to normal values 50 mg/kg: the best protection Balanced the oxidant/anti-oxidant system	(110)
**SYR**	5, 25, 50, 100 5 μM/ for 24 hr	Isolated PBMCs from MI patients/ for 24 hr	Alleviating inflammatory biomarkers such as TNF-α, IL-6, and NO Balance the oxidant/antioxidant system Strong binding affinity between SYR with IL-6, TNF-α, and anti-oxidant markers	(111)
**SYR**	-	HPAECs	Improvement in vascular injury ↓ VCAM-1, ICAM-1, MCP-1, ET-1, E-selectin, iNOS, TNF-α, IL-6, and IL-1β ↑ NO, and nuc-p65 ↑ p-AMPK, IκBα, and cyto-p65 expressions AMPK/NF-κB signaling pathway	(112)
** CGJ**	SYR	H-ECs and aortic rings (3–4 mm width) isolated from Sprague-Dawley	SYR was detected in the serum of PRS, H-ECs, and isolated aortic rings, indicating CPSDS endothelium-dependent vasodilation production of NO, mediated antioxidation-sensitive function of Src kinase, leading to consequential PI3/Akt-mediated phosphorylation of Enos Anti-atherosclerosis efficacy	(138)
**SYR**	Turbidity and Fibrin clot assay 5, 10, and 20 μg/ml for 60 min. Antithrombotic activity: 10, 20, 50, and 100 μg	Male Sprague-Dawley rats treated with FeCl3 induced an arterial thrombosis model	Suppressed the function of procoagulant protease ↓ Clot formation and even destroyed blood clot ↓ Expression of DEP-1, αIIbβ3, and PTP1B ↓ Phosphorylation of kinases involved in epinephrine/collagen-stimulated platelets in mice blood and *in vitro* DEP-1/PTP1B/αIIbβ3/kinases ↓ Thromboembolism and thrombosis	(113)

ALP: alkaline phosphatase; AST: aspartate aminotransferase; ALT: alanine transaminase; CGJ: concord grape juice; CK-MB: creatine kinase-MB; CPSDS: CGJ post-dose supernatant of deproteinated serum; DEP-1: density-enhanced phosphatase-1; eNOS: endothelial nitric oxide synthase; ET-1: endothelin 1; Fn1: fibronectin 1; GGT; gamma-glutamyl transferase; hs-CRP: high-sensitivity C-reactive protein; H-ECs: H_2_O_2_-treated endothelial cells; HDL-C: high-density lipoprotein cholesterol; HPAEC: human pulmonary artery endothelial cells; ICAM-1: intercellular adhesion molecule-1; IL-6: interleukin-6; iNOS: nitric oxide synthase; IP: intraperitoneal; ISO: isoproterenol; IV: intravascular injection; l-NAME: nω-nitro-l-arginine methyl ester; LDH: lactate dehydrogenase; LDL-C: low-density lipoprotein cholesterol, LPL: lipoprotein lipase; LV: left ventricular; LVEF: left ventricular ejection fraction; LVFS: left ventricular fractional shortening; LPS: lipopolysaccharides; MetS: metabolic syndrome; MCP-1: monocyte chemoattractant protein-1; MI: myocardial infarction; NO: nitric oxide; NOx: nitric oxide metabolites; NRF-1: nuclear respiratory factor 1; Nrf2: nuclear factor erythroid 2–related factor 2; PBMCs: peripheral blood mononuclear cells; PRS: post-dose rats; PC: protein carbonyl; PGC-1α: peroxisome proliferator-activated receptor gamma coactivator 1-alpha; PTP1B: protein tyrosine phosphatase-1B; RBC: erythrocyte; SC: subcutaneous injection; STZ: streptozotocin; SYR: syringic acid; TBARS: thiobarbituric acid reactive substances; TG: triglycerides; TGF-β1: transforming growth factor β1; TLR4: toll-like receptor 4; TNF-α: tumor necrosis factor alpha; TPC: total protein content; VCAM-1: vascular cell adhesion molecule 1; VLDL-C: very low-density lipoprotein cholesterol

CPSDS: CGJ post-dose supernatant of deproteinated serum; NO: nitric oxide; p-Akt: phosphorylated Akt; PI3K: phosphoinositide 3-kinase; eNOS: endothelial nitric oxide synthase; LV: Left ventricular; LVEF: left ventricular ejection fraction; LVFS: left ventricular fractional shortening; CK-MB: creatine kinase-MB; LDH: lactate dehydrogenase; PGC-1α: Peroxisome proliferator-activated receptor gamma coactivator 1-alpha; NRF-1: Nuclear respiratory factor 1; Nrf2: nuclear factor erythroid 2–related factor 2; TFAM: transcription factor A mitochondrial; DEP-1: density-enhanced phosphatase-1; PTP1B: protein tyrosine phosphatase-1B

**Table 3:** Protective effects of syringic acid on dyslipidemia

**Source **	**Dose/Concentration**	**Study model**	**Results**	**Ref.**
**SYR**	25 and 50 mg/kg; in diet/ for 7 weeks	Male Wistar rats treated with HFD/ for 7 weeks	Reversed dyslipidemia and improved oxidative stress markers ↓ Inflammatory cytokines, albumin, LDH, and globulin	(118)
**Eutectic mixture of SYR and fenofibric acid**	9.45 mg/kg	Male Wistar rats	Improve dissolution rate, solubility, and lipid-lowering potential ↓ Total cholesterol levels Improve antihyperlipidemic efficacy	(119)
**SYR-SMEDDS**	100 mg/kg; in diet/ for 10 days	Mice treated with HFD/ 21 days	Prolonged t1/2, Tmax, and MRT ↑ Liver accumulation and bioavailability Delayed the elimination of SYR ↓ Lipid profiles and relieved liver steatosis	(120)
**SYR**	50 mg/kg, oral/ for 8 days	Wistar Albino rats treated with APAP 750 mg/kg, IP/ a single dose	↓ TG, Free fatty acids, Phospholipids, and total Cholesterol levels ↑ Vitamin E and Vitamin C Balanced the oxidant/antioxidant system	(121)
**SYR**	25, 50, and 100 mg/kg, oral/ for 8 days	Wistar Albino rats treated with APAP 750 mg/kg; IP/ a single dose	↓ LDL-C, VLDL-C ↑ HDL-C ↓ TG, free fatty acids, phospholipids, and total cholesterol levels in plasma, kidney, and liver tissues	(121)
**SYR**	100-1000 µmol/ml/ for 2 and 10 days	3T3-L1 Preadipocytes	Suppressed the differentiation of 3T3-L1 preadipocytes to mature adipocytes in a dose-dependent manner Prevention of mitotic clonal expansion in the initial stages of differentiation ↑ Lipolysis (glycerol release) ↓ Lipogenesis (triglyceride synthesis) in adipocytes ↓ ROS synthesis	(122)
**SYR**	100–1000 µmol/ml/ for 2 and 10 days	3T3-L1 Preadipocytes	↓ NOX4 expression ↓ ROS synthesis ↑ GSH, CAT, and SOD amounts Balance the oxidant/anti-oxidant system	(123)

APAP: acetaminophen; HDL-C: high-density lipoprotein cholesterol; HFD: high-fat diet; IP: Intraperitoneal; LDL-C: low-density lipoprotein cholesterol; MetS: metabolic syndrome; NOX4: NADPH oxidase 4; ROS: reactive oxygen species; SMEDDS: self-microemulsifying drug delivery system; SYR: syringic acid; TG: triglycerides; VLDL-C: low-density lipoprotein cholesterol

**Table 4:** Protective effects of syringic acid on obesity

**Source**		**Dose/Concentration**	**Study model**	**Results**	**Ref.**
**SYR**	50 mg/kg; in diet/ for 4 weeks After 12 weeks of obesity induction	Mice treated with HFD/ 12 weeks	↓ Lipid biomarkers related to obesity ↓ Weight	(128)
**SYR**	20 mg/kg; in diet/ for 8 weeks	Rats treated with HFD/ 8 weeks	↓ Serum levels of AST and ALT, and suppressed pro-inflammatory cytokines Attenuated histopathological and immunohistochemical alterations Reversed oxidative stress by 82% and replenished antioxidant functions ↑ Gene expressions of Nrf2/heme oxygenase 1 (Nrf2/HO-1)	(129)
**SYR**	0.05%, wt/wt; in diet/ for 16 weeks	C57BL/6J male mice treated with HFD/ 16 weeks	↓ Visceral fat mass, body weight, leptin levels, lipid content of the liver, and insulin resistance ↑ Adiponectin circulation ↑ Expression of fatty acid oxidation genes such as Cpt1, Cpt2, Acsl, and Pparα in the liver ↓ Expression of lipogenic genes, including Srebp-1c, Srebp-2, Cidea, Fasn, Pparγ, and Hmgcr ↑ The activity of fatty acid oxidation enzymes ↑ Lipogenic enzyme activities in liver tissue	(39)
**SYR**	100 mg/kg; in diet/ for 12 weeks	OVX Mice treated with HFD/ 12 weeks	↓ Body fat mass (subcutaneous, visceral, and total fat) ↓ TG	(133)
**SYR**	-	Molecular docking analysis	Higher inhibitory scores of SYR (-5.491 kcal/mol) than Orlistat against obesity	(134)

HFD: high-fat diet; MetS: metabolic syndrome; OVX: ovariectomized; SYR: syringic acid; TG: triglycerides

## Declaration

During the preparation of this work, the author(s) used AI-assisted technologies (Grammarly and iThenticate) to rephrase, reduce plagiarism, and improve language and grammar. After using this tool/service, the author(s) reviewed and edited the content as needed and take(s) full responsibility for the content of the publication.
